# Mapping risk: Spatial analysis of 2,4-D herbicide emissions and colorectal cancer

**DOI:** 10.1016/j.envc.2025.101356

**Published:** 2025-10-27

**Authors:** Danny Baxter, Dan A. Dixon, Andrew J. Morris, Tongzhang Zheng, Yong Zhu

**Affiliations:** a Winthrop P. Rockefeller Cancer Institute, University of Arkansas for Medical Sciences, Little Rock, AR 72205-7199 USA; b Department of Biochemistry and Molecular Biology, College of Medicine, University of Arkansas for Medical Sciences, Little Rock, AR 72205-7199 USA; c Department of Pharmacology and Toxicology, College of Medicine and Central Arkansas Veterans Healthcare System, University of Arkansas for Medical Sciences, Little Rock, AR 72205-7199 USA; d Department of Epidemiology, School of Public Health, Brown University, Providence, RI 02912 USA; e Department of Epidemiology, Fay W. Boozman College of Public Health, University of Arkansas for Medical Sciences, Little Rock, AR 72205-7199 USA

**Keywords:** Colorectal cancer, 2,4-dichlorophenoxyacetic acid, 2,4-D, Herbicides, Air pollution, Spatial epidemiology, Environmental exposure assessment

## Abstract

Colorectal cancer remains a major public health concern in Arkansas and across the United States, disproportionately affecting rural and vulnerable populations. Beyond established lifestyle and genetic factors, environmental exposures, particularly agricultural chemicals, are increasingly recognized as potential contributors. In this ecological study, we screened 133 county-level estimated air pollutants to identify candidates associated with colorectal cancer incidence rates, focusing on Arkansas due to its agricultural economy and widespread herbicide use. Using publicly available environmental and cancer registry data, we applied spatial mapping, correlation tests, linear regression, and spatial regression models. Among screened pollutants, 2,4-Dichlorophenoxyacetic acid (2,4-D), a widely used herbicide, emerged as the top candidate. In Arkansas, 2,4-D emissions showed a strong positive association with colorectal cancer incidence rates (Spearman ρ = 0.338, p = 0.003; linear regression β1 = 6.28, p = 0.001), with high-emission counties aligning spatially with elevated incidence. This relationship was further confirmed in analyses of more than 2,500 U.S. counties, where 2,4-D emissions remained significantly associated with colorectal cancer (linear regression β1 = 5.94, p < 0.001; spatial regression β1 = 2.55, p = 0.001). Spatial regression improved model fit and accounted for geographic autocorrelation in both datasets. These findings suggest a potential association between 2,4-D emissions and colorectal cancer incidence rates, particularly in agricultural regions such as Arkansas. While causal inference is limited by the ecological design, the consistency of results warrants follow-up with individual-level, longitudinal, and mechanistic studies.

## Introduction

Colorectal cancer (CRC) remains a major public health challenge in the United States, with an estimated 107,320 new colon and 46,950 new rectal cancer cases projected in 2025, and about 52,900 deaths Key statistics for colorectal cancer (2024b). CRC is particularly concerning in Arkansas, where incidence and mortality have exceeded national averages for over a decade Arkansas Cancer Facts & Figures (2024a). Alarmingly, CRC rates are rising among younger adults, with recent analyses showing increases in those aged 30–59 (Shiels et al., 2025). While interventions have focused largely on behavioral and genetic risk factors, these trends highlight the need to also consider environmental contributors.

Given rising CRC rates and persistent disparities, attention has turned to environmental exposures as potential contributors alongside diet and genetics. Emerging evidence links air pollutants to CRC risk and worse outcomes; for example, recent large-scale studies have associated higher exposures to PM2.5 and NO2 with increased CRC incidence and mortality (Jiang et al., 2024; Wei et al., 2023). These findings highlight the need to examine environmental factors in CRC patterns. Building on this, we conducted a county-level analysis of air pollutant emissions and CRC incidence, focusing on how geographic variations in exposure may contribute to the CRC burden.

Among environmental pollutants, 2,4-dichlorophenoxyacetic acid (2,4-D) is of particular interest due to its widespread use and potential carcinogenicity. Prior research in humans has focused largely on occupational exposures among farmers and applicators, with mixed findings: some studies report increased risks of non-Hodgkin lymphoma and soft-tissue sarcoma (Kogevinas et al., 1995; Smith et al., 2017), some found an increased risk of mortality in rectal cancer (Schreinemachers, 2000), while others suggest no association or even an inverse relationship with CRC (Burns et al., 2011; Lee et al., 2007). The International Agency for Research on Cancer classifies 2,4-D as “possibly carcinogenic to humans” (Group 2B) based on limited evidence. However, little is known about the health effects of chronic, low-level environmental exposure in the general population, especially through airborne herbicide emissions in rural areas. To address this knowledge gap, we systematically screened county-level air pollutant emissions data, focusing specifically on whether estimated airborne 2,4-D emissions are associated with CRC incidence across the United States, using publicly available data and spatial epidemiologic methods.

## Methods

### Data sources and processing

We used publicly available data for all analyses. Environmental data came from the U.S. EPA’s 2020 Air Toxics Screening Assessment (AirToxScreen), based on the 2020 National Emissions Inventory (NEI) (Bureau, 2021), providing county-level pollutant emission estimates. CRC incidence rates (age-adjusted per 100,000, invasive cases, all races, both sexes, 2017–2021 average) were obtained from the NCI’s State Cancer Profiles (SCP) database State Cancer Profiles (2024c). Confounding data on poverty and uninsured rates were obtained from the United States Census Bureau’s American Community Survey database and pulled into R using API (Bureau, 2021) Datasets were cleaned and merged using standardized county identifiers (FIPS codes, GEOID). Pollutants with missing or zero values in ≥ 38 of Arkansas’s 75 counties were excluded to ensure sufficient variability. For pollutants reported by multiple emission sources within a county, emissions were averaged to a single value. County names were standardized across datasets for consistency.

### Statistical analysis

We applied multiple statistical approaches to assess associations between air pollutant emissions and CRC incidence rates. As Shapiro–Wilk tests indicated non-normality, initial analyses used nonparametric methods, calculating Spearman’s rank correlations between each pollutant and CRC incidence rates, with p-values adjusted using the Benjamini–Hochberg false discovery rate correction. Given strong initial signals for 2,4-D, we next employed linear regression models, first in Arkansas counties and then extended to all U.S. counties with available data. After conducting the full dataset regression analyses, we then stratified counties by rural-urban status, as defined by the USDA’s 2023 Rural-Urban Continuum Codes (RUCC) and performed additional regression analyses on the stratified data. Finally, to account for spatial autocorrelation, we applied spatial regression models on the national dataset.

### Geographic analysis

Geospatial data for the spatial error model, including shapefiles, neighborhood structure, and spatial weight matrix—were generated entirely in R, using only EPA AirToxScreen and CDC State Cancer Profiles as external sources. U.S. county shapefiles were obtained via the tigris package and merged with study data on 2,4-D emissions and CRC incidence rates using FIPS codes. A first-order queen contiguity neighborhood structure (poly2nb()) and corresponding spatial weights matrix (nb2listw()) were constructed for spatial modeling.

### Software

All data cleaning, statistical analyses, and visualizations were conducted in Microsoft Excel (Version 2503) and R (v4.4.1) within RStudio (v2024.12.1). Packages used include readxl, lmtest, dplyr, tigris, sf, spdep, spatialreg, ggplot2, e1071, tidycensus, and tidyr.

## Results

The AirToxScreen dataset included 193 air pollutants in Arkansas. To ensure sufficient variability and statistical power, we excluded pollutants missing or zero in ≥ 38 of 75 counties, leaving 133 for analysis. Spearman’s rank correlations between each pollutant and CRC incidence rates identified four candidates (ρ > 0.1): 2,4-D, trifluralin, antimony, and diesel particulate matter. A complete list of pairwise Spearman’s rank correlation coefficient test results can be found in Supplementary Table 1. Antimony and diesel particulate matter were excluded due to non-significant p-values. Trifluralin initially showed a moderate correlation (ρ = 0.323, p = 0.005) in Arkansas, but further national-level analyses revealed a weak and inconsistent association, and it was dropped from further consideration.

This left 2,4-D solely showing a robust correlation with CRC incidence rates in Arkansas (ρ = 0.337, p = 0.003; FDR-adjusted p = 0.007). Given the large number of pairwise correlation tests conducted, Benjamini-Hochberg false discovery rate (FDR) adjusted p-values were calculated to account for multiple comparisons. The FDR correction for 2,4-D remained significant at 0.007. A complete list of pollutant correlations with adjusted and unadjusted p-values can be found in Supplementary Table 2. To visually explore spatial patterns in environmental exposure and CRC disease burden, county-level choropleth maps of Arkansas were created to display the estimated distribution of 2,4-D emissions in tons and age-adjusted CRC incidence rates (Fig. 1). Choropleth maps revealed clustering of both 2,4-D emissions and CRC incidence rates in eastern Arkansas, particularly the agriculturally intensive Delta region, a geographic area known for sociodemographic disparity and strong agricultural importance.

Before implementing further analyses, a log transformation was indicated to improve the distribution of 2,4-D emissions data. Ultimately, a log10(*x* + 1) transformation was chosen and applied to the variable. After log transformation, Spearman’s correlation coefficient for 2,4-D emissions and CRC incidence rates by county in Arkansas shifted slightly, returning a ρ of 0.338 and an unadjusted p-value of 0.003, confirming continued robustness. A complete list of the log-transformed pollutant emission estimates can be found in Supplementary Table 3.

To quantify this relationship, we performed linear regression on Arkansas counties (*N* = 75) (Fig. 2), finding that each one-unit increase in log-transformed 2,4-D emissions corresponded to 6.28 additional CRC cases per 100,000 (p = 0.001, R^2^ = 0.13). The county-level data were then stratified by RUCC rural-urban status for additional regression analysis. Among rural Arkansas counties (*N* = 58), each one-unit increase in log-transformed 2,4-D emissions was associated with 6.38 additional CRC cases per 100,000 (*p* = 0.003, R^2^ = 0.13). Among urban Arkansas counties (*N* = 17), the increase was 5.95 additional CRC cases per 100,000 (*p* = 0.04, R^2^ = 0.25). While the explanatory power of the models is limited, such findings remain valuable as early evidence of potential associations that merit further investigation with more granular data.

To strengthen model power and examine the association at a larger scale, we expanded the dataset to include 2555 U.S. counties (~80 % of all counties) with available data from EPA AirToxScreen and CDC State Cancer Profiles (see Limitations for data gaps). A linear regression on the national dataset (Fig. 3) estimated an increase of 5.94 CRC cases per 100,000 per log-unit increase in 2,4-D emissions (SE = 0.60, p < 0.001), explaining 3.8 % of the variance (R^2^ = 0.04) with a residual standard error of 9.35. Although modest, this association was statistically significant and likely reflects the influence of unmeasured confounders at the national level. The national data was also stratified by RUCC and reanalyzed. Among rural U.S. counties (*N* = 1496), each one-unit increase in log-transformed 2,4-D emissions was associated with 5.37 additional CRC cases per 100,000 (*p* = <0.001, R^2^ = 0.03). Among urban U.S. counties (*N* = 1059), the increase was 4.76 CRC cases per 100,000 (*p* = <0.001, R^2^ = 0.03). R^2^ values continue to be modest, underscoring the exploratory rather than confirmatory nature of this analysis.

The spatial nature of county-level data raises concerns about violating the independence assumption of linear regression, as spatial correlation can bias estimates. Moran’s I test indicated strong spatial autocorrelation in the ordinary least squares (OLS) model residuals (*I* = 0.33, *p* < 0.001), indicating a violation of independence in the OLS model and supporting the use of a spatial regression model for better fit. Multiplier diagnostics supported a spatial error specification (robust LM error *p* < 0.001; robust LM lag not significant). The spatial error model significantly improved fit over the OLS model (AIC reduced from 18,680 to 18,178; log likelihood increased from −9337 to −9084). The spatial error model confirmed a significant positive association between log-transformed 2,4-D emissions and CRC incidence rates (β_1_ = 2.55, SE = 0.80, p = 0.001), indicating 2.55 additional CRC cases per 100,000 per log-unit increase in emissions. The model also revealed moderate spatial dependence (λ = 0.53, p < 0.001), indicating that nearby counties tended to have similar unexplained variation in CRC incidence rates. Though each county is its own distinct geographic unit, areas that are in spatial proximity to each other will often have shared contextual environments that do not stop at county lines. Important shared factors such as overlapping agricultural zones, similar socioeconomic profiles, regional environmental or agricultural policies, and infrastructure patterns can influence both environmental exposure and disease risk. This spatial error correlation emphasizes the importance of regional clustering effects that cannot be fully captured by basic regression models and supports the use of spatial modeling to account for these effects. It should still be noted, however, that the overall explanatory power of the model remains modest, consistent with exploratory associations.

Many strong risk factors in CRC incidence rates have been extensively studied, especially socioeconomic and behavioral risk factors. To account for these risk factors as potential confounders in our model, we utilized the 2021 American Community Survey (ACS) 5-year estimate data from the United States Census Bureau to incorporate the percentage variables “ % without health insurance coverage” (DP02_0096PE) and “ % of population below poverty level in the past 12 months” (DP03_0128PE) (Bureau, 2021). These variables were joined to the existing dataset by GEOID and used to reanalyze the SEM. In the adjusted model, the positive association between 2,4-D emissions and CRC incidence rates persisted (β = 2.81, SE = 0.74, *p* < 0.001); % of population below poverty level in the past 12 months was strongly associated with higher CRC incidence rates (β = 0.40, SE = 0.03, *p* < 0.001), while % without health insurance coverage was not significant. Adjustment reduced residual spatial autocorrelation (λ from 0.53 to 0.46) and improved model fit (AIC from 18,178 to 18,028). While adjustment improved model fit and affirmed the persistence of the association between 2,4-D and CRC incidence rates, the explanatory power of the model remains modest throughout all four models. Accordingly, these findings should be interpreted as exploratory and hypothesis-generating.

## Discussion

This exploratory study aimed to identify and quantify associations between modeled air pollutant emissions and CRC incidence rates at the county level in Arkansas and nationwide. We found a consistent, modest but statistically significant association between 2,4-Dichlorophenoxyacetic acid (2,4-D) emissions and CRC incidence rates, with overlapping hotspots observed in eastern Arkansas. The relationship held under multiple statistical models and was further supported at the national level, with spatial regression confirming the association while accounting for geographic dependence. Though this study and analysis are exploratory in nature and therefore unable to definitively prove an association, these findings are noteworthy, hypothesis-generating, and indicate a need for more direct study.

Understanding these findings requires context on 2,4-D’s toxicological profile. The Agency for Toxic Substances and Disease Registry (ATSDR) identifies 2,4-D as a widely used synthetic herbicide, primarily encountered through inhalation, ingestion, or skin contact in agricultural settings. Although rapidly excreted, concerns remain about the effects of chronic or high-level exposures, which are still poorly understood. Unsurprisingly, the eastern Arkansas Delta, a major agricultural region, accounted for much of the state’s 2,4-D emissions.

The carcinogenic potential of 2,4-D remains controversial. The International Agency for Research on Cancer (IARC) classifies it as “possibly carcinogenic to humans” (Group 2B) International Agency for Research on Cancer (2015) based on limited animal evidence and inadequate human data, noting oxidative stress and immunosuppression as plausible mechanisms. In contrast, the U.S. EPA has deemed evidence insufficient to establish a causal link to cancer Environmental Protection Agency (2007). Since the 2007 ruling, a specific formulation of 2,4-D known as 2,4-D choline, used in the commercial herbicides Enlist One and Enlist Duo, has undergone repeated scrutiny and mitigation in relation to the Endangered Species Act (Agency, 2025). In 2023, EPA’s official biological opinion on the Enlist products determined that the products, when used as labeled, “are not likely to jeopardize any listed species or adversely modify their critical habitats” (Agency, 2023). Despite this ruling, the impact of these herbicides on the environment remains ongoing, and further developments are expected to continue. A 2013 systematic review found a lack of evidence for DNA-damage carcinogenicity but left open the possibility of other forms of carcinogenicity depending on the level of exposure (von Stackelberg, 2013). The observed association may reflect indirect pathways rather than direct carcinogenicity. Given the ongoing debate over 2,4-D’s carcinogenic potential, this relationship warrants further investigation to clarify its role in cancer development, whether through direct or indirect mechanisms. Evolving regulatory assessments have practical implications for how 2,4-D emissions and exposure patterns evolve over time. Regulatory changes (e.g., new restrictions on use, new formulations, or mitigations through the Endangered Species Act) can impact environmental release through changes to where and how much 2,4-D is applied in the U.S. Long-term monitoring and modeling efforts will be essential to the proper evaluation of how regulatory dynamics shape changes in ambient concentrations and human exposures.

Though 2,4-D’s direct carcinogenicity remains contested, indirect pathways to carcinogenesis, including for CRC, show biological plausibility in some studies. One possible mechanism involves the gut microbiome, increasingly implicated in CRC risk and progression. Prior in vivo findings showed that low-dose 2,4-D exposure disrupted gut microbiota and host metabolism in mice, while human studies have associated microbiome dysbiosis with CRC tissue and treatment outcomes (Tu et al., 2019). Another study in rats found negative impacts on the esophagus and large and small intestines after exposure to concentrations of 2,4-D similar to those typically used in agriculture (Mariotti et al., 2022). Another possible mechanism is through the induction of oxidative stress. A 2021 study found 2,4-D can trigger oxidative stress responses and unfolded protein stress, which are mechanisms that can contribute to tumor-promoting environments (Mesnage et al., 2021). Though these studies do not directly address CRC in humans, the mechanisms studied provide biologically plausible pathways through which 2,4-D exposure could contribute to biological environments that might increase the risk for cancer, including CRC. Nonetheless, the ecological design of our study and absence of individual-level exposure or biomarker data require further research to clarify the prevalence of these pathways in humans.

Taken together, our results suggest an intriguing correlation that warrants further investigation into whether and how 2,4-D might act as an environmental risk factor for CRC and potentially other cancers. Because CRC risk arises from a complex interplay of genetic, behavioral, and environmental factors, our findings point to the need for broader exploration of how agricultural pollutants contribute to regional cancer disparities.

The association we observed also raises public health and environmental justice concerns. Rural communities already face higher baseline cancer risks, limited preventive care, and greater exposure to agricultural chemicals. In our study, stratification by rural-urban status showed higher CRC incidence rates and 2,4-D estimated emissions in rural counties than in their urban counterparts on average. Targeted environmental monitoring, improved cancer screening in high-exposure areas, and stricter regulation of herbicide use could help mitigate these risks. Collaborative efforts among environmental agencies, public health professionals, and policymakers are essential to protect vulnerable populations from preventable exposures.

Although poverty proved to be a strong predictor of CRC incidence rates in our model, the lack of a significant association for health insurance coverage was somewhat unexpected. This may suggest that insurance status itself is highly correlated with poverty and, therefore, the presence of one masks the other. Additionally, it is possible that poverty captures more direct pathways of disadvantage (e.g., limited preventive care, poorer diet quality, environmental exposures, etc.) or that unmeasured factors such as coverage quality, Medicaid expansion, and/or healthcare infrastructure contribute to the complex relationship between insurance coverage and CRC risk.

This exploratory analysis has several limitations. While our analysis was initiated in Arkansas to explore potential county-level associations in a largely rural state with substantial agricultural activity, we expanded the analysis to all U.S. counties to determine whether or not these patterns hold at the national level. Still, regional differences in agricultural practices, crop types, and pesticide usage may limit generalization to less agricultural areas of the country. Next, data coverage was incomplete in some states. Specifically, county-level CRC incidence rates were unavailable for Indiana and Kansas, and 2,4-D emissions data were not reported for Alaska, Hawaii, Indiana, or Kansas, limiting full national representation.

Further, there is a temporal mismatch between the exposure estimates (2020) and CRC incidence rate data (2017–2021). Perfect temporal alignment between environmental data and cancer outcomes is difficult to achieve due to the long latency between exposure and cancer diagnosis. Therefore, our objective, rather than to infer direct causality, was to identify spatial patterns that may suggest areas of concern. Given that 2,4-D has been commercially available and widely used for many decades, usage patterns are likely relatively stable over time. Therefore, we believe the 2020 AirToxScreen estimates serve as a reasonable proxy for exposure conditions in previous years. Still, this temporal mismatch necessitates cautious interpretation, and true causality cannot be determined by this data alone. Additionally, our analysis focused on “2,4-D, Salts and Esters” as defined in the EPA AirToxScreen dataset. These are commonly applied forms of 2,4-D but may differ in environmental and toxicological behavior from other formulations, potentially underestimating total population exposure.

Next, though the use of a spatial regression model helps to address geographic autocorrelation, the model presented here does not fully capture all of the potential hierarchical or contextual effects that could arise from deeper factors. Nested influences could be addressed through the addition of spatial covariates or a multilevel framework, helping to further refine exposure-disease pathways. This analysis was designed for broad identification of spatial patterns using existing, publicly available data; however, future studies integrating more granular covariates are needed to definitively establish associations. Additionally, important behavioral and lifestyle factors strongly associated with CRC risk, such as smoking prevalence, obesity rates, dietary patterns, and CRC screening rates, were not available to be included in this analysis. These factors have been repeatedly established and reaffirmed as determinants of CRC incidence rates. Their geographic variation across counties in the U.S. has the potential to influence the associations observed here. Though our study adjusted for poverty and health insurance coverage as key socioeconomic indicators, the absence of other variables creates a possibility for residual confounding. Future analyses should incorporate an expanded list of potential confounding variables to more fully account for variation in CRC risk across U.S. counties and strengthen causal inference.

Finally, although this study leveraged EPA-modeled air pollutant emissions data to examine potential associations between 2,4-D exposure and CRC incidence rates, it is important to recognize the limitations of using airborne estimates as a proxy for total human exposure. Multiple pathways exist for exposure to herbicides like 2,4-D, and they may impact the body differently. Specifically, ingestion, particularly through contaminated drinking water and food grown in treated soil, may represent more significant exposure pathways than inhalation in the general population. Therefore, while our findings suggest a spatial correlation between airborne 2,4-D emissions and CRC incidence rates, future studies incorporating environmental measurements from water and soil, as well as human biomonitoring data, are needed to more comprehensively assess exposure and confirm this relationship. Additionally, proximity to areas of heavy agricultural activity is an important factor that requires deeper consideration and would be better defined at a higher scale than our county-level data. Future research should incorporate 2,4-D exposure data at a more granular level to better approximate exposure at the individual level.

Nevertheless, this study is among the first to focus on ambient 2,4-D exposure at the community level rather than the occupational level and systematically examine the association between county-level airborne 2,4-D emissions and CRC incidence rates across the U.S., using publicly available environmental and cancer surveillance data and robust spatial models. We demonstrate that 2,4-D emissions spatially overlap with CRC hotspots, even after accounting for spatial dependence, underscoring the need to explore possible environmental contributors to CRC risk. By focusing on ambient, community-level exposures rather than solely occupational cohorts, this work establishes a basis for addressing an important gap in understanding the environmental determinants of cancer disparities and underscores the value of spatial epidemiology in guiding targeted interventions. While causality cannot be established, these findings support integrating environmental health into cancer surveillance and prevention efforts, particularly in high-exposure rural and agricultural communities. Policymakers and public health agencies should prioritize enhanced cancer screening, improved environmental monitoring, and evaluation of safer agricultural practices in these regions. Future research should aim to validate these associations with individual-level exposure data, directly measured confounders, and longitudinal data to confirm the associations found in the current study. Further, future research should elucidate biological pathways, such as epigenetic modifications and microbiome disruption, through which pollutants like 2,4-D may influence cancer risk. Finally, more refined exposure modeling and data at finer geographic scales, alongside consideration of genetic, behavioral, and sociodemographic factors, could enable more effective and targeted prevention strategies.

## Supplementary Material

Suppl Table 1-3

Supplementary material associated with this article can be found, in the online version, at doi:10.1016/j.envc.2025.101356.

## Figures and Tables

**Fig. 1.**
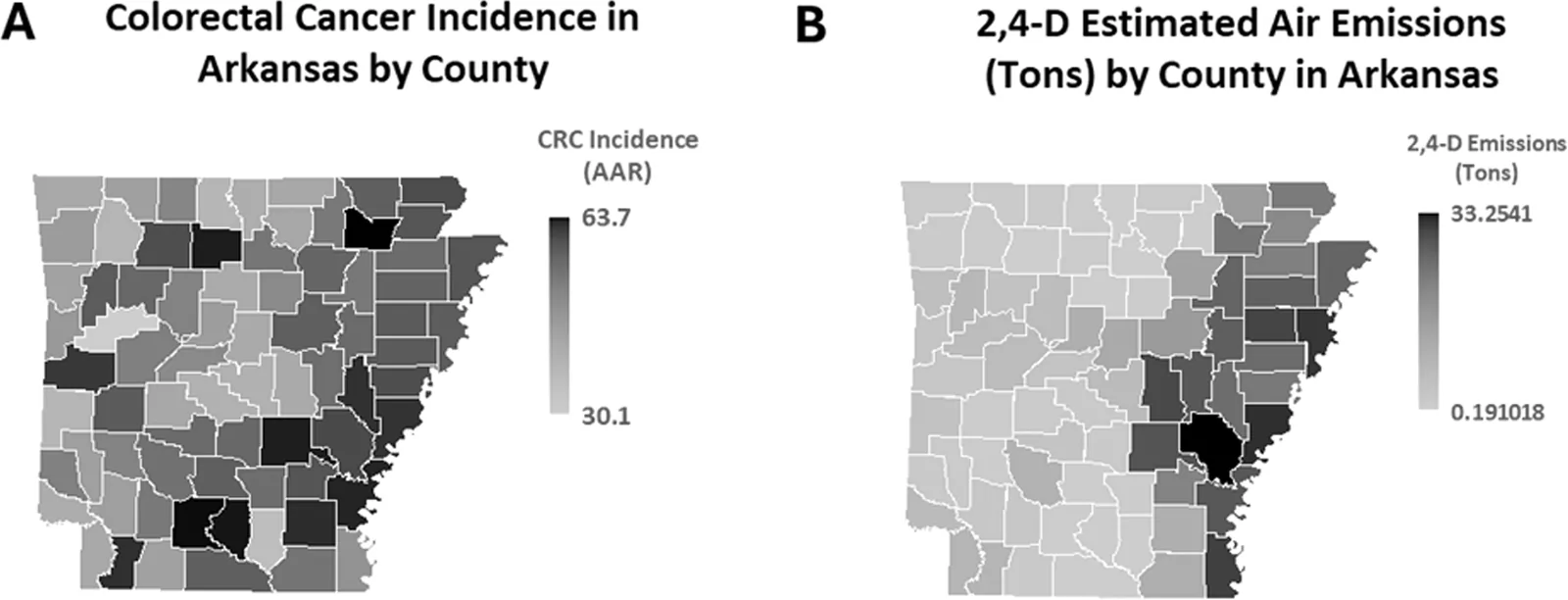
Choropleth maps of age-adjusted colorectal cancer incidence rates and 2,4-D estimated air emissions in tons by county in Arkansas. Fig. 1A demonstrates scattered incidence rates across the state, with some clustering of higher incidence counties in the eastern region. Similarly, Fig. 1B shows strong clustering of 2,4-D emission estimates in the eastern region of the state, known for its agricultural importance. Spatial overlap may warrant further investigation into associations between 2,4-D and CRC incidence rates, though visual inspection alone cannot determine any link.

**Fig. 2.**
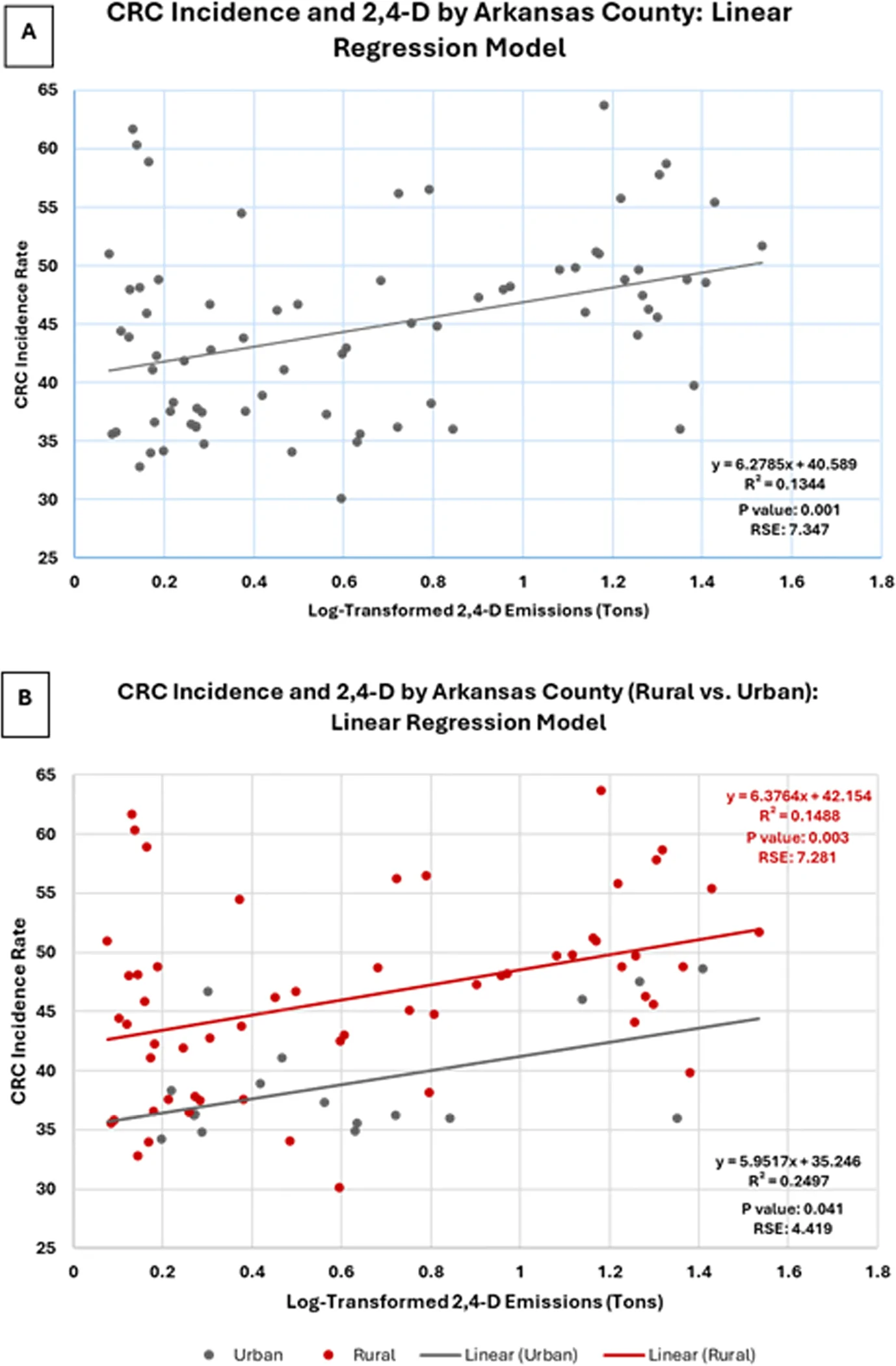
Linear regression models of age-adjusted colorectal cancer incidence and log-transformed 2,4-D emissions (tons) across all Arkansas counties and stratified by rural-urban status. County-level analysis (*N* = 75) indicated a positive correlation between 2,4-D emissions and CRC incidence rates, with each one-unit increase in 2,4-D emissions associated with 6.28 additional CRC cases per 100,000 (*p* = 0.001, R^2^ = 0.13, SE = 1.87, residual standard error [RSE] = 7.35) (Fig. 2A). In rural counties (*N* = 58), 2,4-D emissions were correlated with an additional 6.38 cases per 100,000 (*p* = 0.003, R^2^ = 0.13, SE = 2.04, RSE = 7.28); in urban counties (*N* = 17), the association was 5.95 additional cases per 100,000 (*p* = 0.04, R^2^ = 0.20, SE = 2.66, RSE = 4.42) (Fig. 2B). While statistically significant, modest R^2^ values indicate limited explanatory power. Incidence rates represent a 5-year average (2017–2021).

**Fig. 3.**
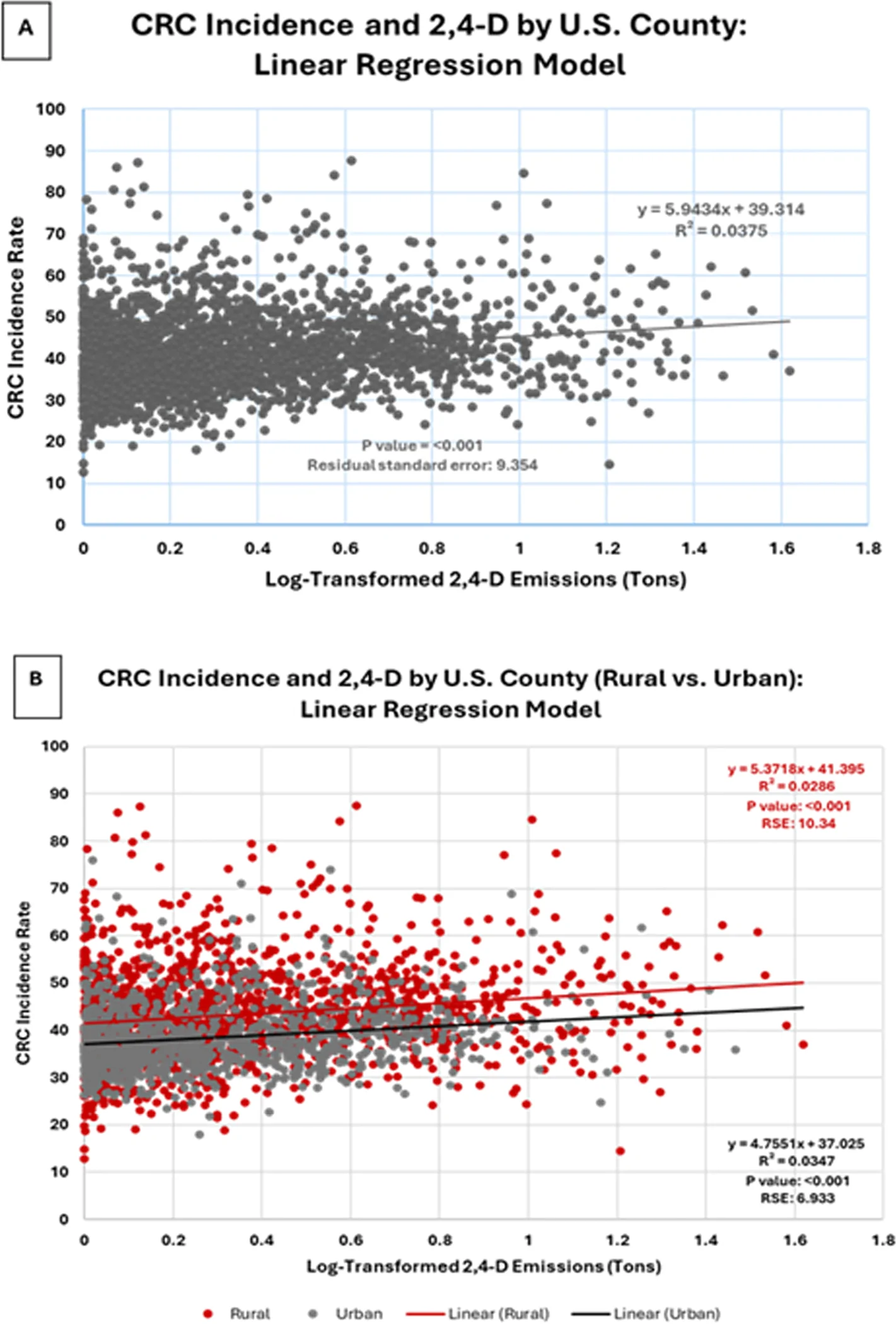
Linear regression model of age-adjusted colorectal cancer incidence and log-transformed 2,4-D emissions (tons) by 2555 U.S. counties (~80 % of all counties) and stratified by rural-urban status. County-level analysis (*N* = 2555) (Fig. 3A) indicated a positive correlation between 2,4-D emissions and CRC incidence rates, with each one-unit increase in 2,4-D emissions associated with 5.94 additional CRC cases per 100,000 (*p* < 0.001, R^2^ = 0.038, SE = 0.60, RSE = 9.35). In rural counties (*N* = 1496), 2,4-D emissions were correlated with an additional 5.37 cases per 100,000 (*p* < 0.001, R^2^ = 0.029, SE = 0.81, RSE = 10.34); in urban counties (*N* = 1059), the association was 4.76 additional cases per 100,000 (*p* < 0.001, R^2^ = 0.035, SE = 0.77, RSE = 6.93) (Fig. 3B). While statistically significant, modest R^2^ values indicated limited explanatory power. Rates represent a 5-year average (2017–2021); data were not available for all states.

## Data Availability

Data will be made available on request.
