# Supplementary material for: Mapping risk: Spatial analysis of 2,4-D herbicide emissions and colorectal cancer
Source: Environ Chall (Amst). Author manuscript; Available in PMC 2026 Oct 8. (PMC13645371; doi:10.1016/j.envc.2025.101356)
Supplement: Supplementary file 1 [file NIHMS2207024-supplement-Suppl_Table_1-3.docx]

**Supplementary Table 1: Top 20 Spearman’s correlation coefficient test results for EPA AirToxScreen and colorectal cancer incidence by county in Arkansas.** Sorted by highest to lowest Spearman’s σ with unadjusted p-values.

| *Pollutant* | *Spearman’s ρ* | *P-value* |
| --- | --- | --- |
| ***2,4-Dichlorophenoxy Acetic Acid*** | 0.337 | 0.003 |
| ***Trifluralin*** | 0.323 | 0.005 |
| ***Antimony*** | 0.188 | 0.106 |
| ***Diesel PM*** | 0.124 | 0.287 |
| ***Arsenic*** | 0.083 | 0.480 |
| ***Methanol*** | 0.079 | 0.502 |
| ***Manganese*** | 0.077 | 0.513 |
| ***Lead*** | 0.062 | 0.598 |
| ***Chromic Acid (VI)*** | 0.020 | 0.899 |
| ***Decachlorobiphenyl (PCB-209)*** | 0.020 | 0.899 |
| ***Nickel*** | 0.012 | 0.915 |
| ***Cumene*** | -0.026 | 0.824 |
| ***Chromium (VI)*** | -0.034 | 0.773 |
| ***2,4-Toluene Diisocyanate*** | -0.047 | 0.766 |
| ***Cadmium*** | -0.063 | 0.589 |
| ***Propionaldehyde*** | -0.082 | 0.482 |
| ***Carbaryl*** | -0.093 | 0.428 |
| ***Beryllium*** | -0.095 | 0.417 |
| ***Ethyl Acrylate*** | -0.128 | 0.273 |

**Supplementary Table 2: Top 20 Spearman’s correlation coefficient AirToxScreen pollutants before and after Benjamini-Hochberg false discovery rate adjustment.** Sorted by BH FDR P-value.

| *Pollutant* | *Unadjusted P-value* | *BH FDR P-value* |
| --- | --- | --- |
| ***2,4-Dichlorophenoxy Acetic Acid*** | 0.003135677 | 0.006985138 |
| ***Trifluralin*** | 0.004659263 | 0.009248985 |
| ***Antimony*** | 0.105662569 | 0.144877543 |
| ***Ethyl Acrylate*** | 0.273271397 | 0.308009287 |
| ***Diesel PM*** | 0.287305766 | 0.321106445 |
| ***Beryllium*** | 0.416805636 | 0.46195958 |
| ***Carbaryl*** | 0.427819734 | 0.470248138 |
| ***Arsenic*** | 0.479608064 | 0.521714824 |
| ***Propionaldehyde*** | 0.482488145 | 0.521714824 |
| ***Methanol*** | 0.502283434 | 0.53873949 |
| ***Manganese*** | 0.513276844 | 0.546126562 |
| ***Cadmium*** | 0.588816827 | 0.621528873 |
| ***Lead*** | 0.598400857 | 0.626671764 |
| ***2,4-Toluene Diisocyanate*** | 0.766400561 | 0.796338083 |
| ***Chromium (VI)*** | 0.772872589 | 0.796837631 |
| ***Cumene*** | 0.824351544 | 0.843375041 |
| ***Chromic Acid (VI)*** | 0.898667208 | 0.905475293 |
| ***Decachlorobiphenyl (PCB-209)*** | 0.898667208 | 0.905475293 |
| ***Nickel*** | 0.915298254 | 0.915298254 |

**Supplementary Table 3: 2,4-Dichlorophenoxyacetic acid estimated emissions by county in Arkansas before and after Log10(x+1) transformation.** Sorted alphabetically by county.

| *County Name* | *2,4-D Estimated Emissions (Tons)* | *Log-Adjusted 2,4-D Estimated Emissions (Tons)* |
| --- | --- | --- |
| ***Arkansas*** | 33.254 | 1.535 |
| ***Ashley*** | 5.432 | 0.808 |
| ***Baxter*** | 0.214 | 0.084 |
| ***Benton*** | 0.866 | 0.271 |
| ***Boone*** | 0.758 | 0.245 |
| ***Bradley*** | 0.394 | 0.144 |
| ***Calhoun*** | 0.375 | 0.138 |
| ***Carroll*** | 0.633 | 0.213 |
| ***Chicot*** | 23.048 | 1.381 |
| ***Clark*** | 4.647 | 0.752 |
| ***Clay*** | 13.541 | 1.163 |
| ***Cleburne*** | 0.492 | 0.174 |
| ***Cleveland*** | 0.447 | 0.161 |
| ***Columbia*** | 0.922 | 0.284 |
| ***Conway*** | 5.253 | 0.796 |
| ***Craighead*** | 12.763 | 1.139 |
| ***Crawford*** | 2.641 | 0.561 |
| ***Crittenden*** | 24.606 | 1.408 |
| ***Cross*** | 22.192 | 1.365 |
| ***Dallas*** | 0.328 | 0.123 |
| ***Desha*** | 19.210 | 1.306 |
| ***Drew*** | 5.167 | 0.790 |
| ***Faulkner*** | 5.976 | 0.844 |
| ***Franklin*** | 3.811 | 0.682 |
| ***Fulton*** | 0.817 | 0.259 |
| ***Garland*** | 0.869 | 0.272 |
| ***Grant*** | 1.005 | 0.302 |
| ***Greene*** | 11.076 | 1.082 |
| ***Hempstead*** | 1.402 | 0.381 |
| ***Hot Spring*** | 1.832 | 0.452 |
| ***Howard*** | 0.872 | 0.272 |
| ***Independence*** | 6.986 | 0.902 |
| ***Izard*** | 0.510 | 0.179 |
| ***Jackson*** | 17.044 | 1.256 |
| ***Jefferson*** | 19.858 | 1.319 |
| ***Johnson*** | 2.155 | 0.499 |
| ***Lafayette*** | 4.284 | 0.723 |
| ***Lawrence*** | 14.209 | 1.182 |
| ***Lee*** | 13.787 | 1.170 |
| ***Lincoln*** | 12.106 | 1.117 |
| ***Little River*** | 3.324 | 0.636 |
| ***Logan*** | 2.929 | 0.594 |
| ***Lonoke*** | 21.450 | 1.351 |
| ***Madison*** | 0.577 | 0.198 |
| ***Marion*** | 0.474 | 0.168 |
| ***Miller*** | 4.253 | 0.720 |
| ***Mississippi*** | 17.104 | 1.258 |
| ***Monroe*** | 15.576 | 1.219 |
| ***Montgomery*** | 0.540 | 0.188 |
| ***Nevada*** | 1.384 | 0.377 |
| ***Newton*** | 0.191 | 0.076 |
| ***Ouachita*** | 0.350 | 0.130 |
| ***Perry*** | 1.926 | 0.466 |
| ***Phillips*** | 25.807 | 1.428 |
| ***Pike*** | 0.521 | 0.182 |
| ***Poinsett*** | 17.470 | 1.266 |
| ***Polk*** | 2.057 | 0.485 |
| ***Pope*** | 2.952 | 0.597 |
| ***Prairie*** | 18.920 | 1.299 |
| ***Pulaski*** | 3.266 | 0.630 |
| ***Randolph*** | 8.062 | 0.957 |
| ***Saline*** | 0.941 | 0.288 |
| ***Scott*** | 1.355 | 0.372 |
| ***Searcy*** | 0.459 | 0.164 |
| ***Sebastian*** | 1.624 | 0.419 |
| ***Sevier*** | 1.016 | 0.305 |
| ***Sharp*** | 0.319 | 0.120 |
| ***St. Francis*** | 15.898 | 1.228 |
| ***Stone*** | 0.267 | 0.103 |
| ***Union*** | 0.394 | 0.144 |
| ***Van Buren*** | 0.236 | 0.092 |
| ***Washington*** | 0.660 | 0.220 |
| ***White*** | 8.379 | 0.972 |
| ***Woodruff*** | 18.037 | 1.280 |
| ***Yell*** | 3.046 | 0.607 |
